# Nanosecond modulation of thermal emission

**DOI:** 10.1038/s41377-019-0179-1

**Published:** 2019-07-24

**Authors:** Daniel Wasserman

**Affiliations:** Department of Electrical and Computer Engineering, University of Texas Austin, Austin, TX 78758 USA

**Keywords:** Mid-infrared photonics, Optoelectronic devices and components

## Abstract

Femtosecond laser pulses are used to modulate the thermal emission from semiconductor materials at the nanosecond timescale. A visible-frequency laser photoexcites energetic free carriers in intrinsic Si and GaAs wafers. As these free carriers return to equilibrium, they not only emit thermal radiation on a picosecond time scale but also modulate the semiconductor thermal emission on a nanosecond to microsecond time scale, offering a novel route towards ultrafast infrared optical pulses.

The field of mid-infrared (mid-IR, 2–30 μm) photonics continues to garner significant research interest, in large part due to a wide range of sensing and security applications arising from the mid-IR’s role as the spectral home to a broad range of vibrational and rotation molecular absorption resonances, as well as the peak thermal emission from nearly any finite-temperature object, such as living organisms, electronic components, and the products of chemical reactions. The drive to probe the former and observe or image the latter has generated, over the past decades, a wealth of new photonic and optoelectronic devices for the generation, detection, and manipulation of mid-IR light.

Much of this effort has resulted in the demonstration and development of new types of mid-IR light sources capable of high optical power and broad wavelength flexibility, in particular the quantum and interband cascade lasers^1,2^, which over the past decades have moved from initial laboratory demonstrations to commercial availability with impressive speed. These semiconductor sources have driven a range of sensing technologies, offering narrow-linewidth emission ideally suited for measuring distinct gas absorption lines across much of the mid-IR^3^. However, there is increasing interest in measuring the temporal response of materials and devices at mid-IR frequencies, for which cascade lasers might not be ideally suited. This interest has driven the development of a new class of nonlinear fiber-based light sources offering sub-picosecond pulses of increasingly longer wavelengths^4^. There remains, however, much interest in alternative mechanisms for inexpensive, ultrafast mid-IR sources, and recent work has suggested, somewhat surprisingly, that thermal emission may offer a potential route for the development of such sources.

Thermal emission from heated bodies follows Planck’s Law of thermal radiation, which governs the spectral shape and intensity of the radiation emitted from a blackbody as a function of temperature, with peak thermal emission following Wien’s Law and moving to shorter wavelengths (and growing in intensity) with increasing temperature. The emission from a blackbody (a surface capable of perfect absorption of light across the entire electromagnetic spectrum) can be spectrally modulated by engineering the absorption spectrum and thus, by Kirchhoff’s Law, the spectral emissivity of the surface^5^. Thermal processes in solid-state materials often occur on time scales much longer than their electronic or optical counterparts. While dependent on the size (heat capacity) and thermal conductivity of the material, thermal energy typically dissipates on micro-second or milli-second time scales, and thus it would seem unrealistic to think that thermal emission could be used as a source of mid-IR light at nanosecond (or even faster) time scales.

Recent work by Xiao and coauthors^6^, however, has demonstrated that thermal emission from a semiconductor material can be controlled on timescales as short as a few picoseconds using ultrafast short wavelength optical pulses. In this work, the authors excite intrinsic semiconductor materials with above-bandgap femtosecond pulses, generating substantial carrier concentrations at energies well above the semiconductor bandedges (Fig. 1a). These highly energetic free carriers are often referred to as “hot carriers” with characteristic temperatures (~4000 K) much greater than that of the semiconductor lattice (~500 K). While these carriers quickly (<3 ps) return to the bandedge of the semiconductor via phonon and Auger scattering processes, they will, while “hot”, contribute a signal to the total thermal emission from the system on the time scale associated with their return to the bandedge and at a wavelength associated with their elevated temperature. Upon their return to the bandedge (Fig. 1b), these carriers can then be treated as photoexcited free carriers, which will contribute to the semiconductor permittivity according to the Drude model. This change in the permittivity alters the emissivity of the semiconductor materials (which are held at fixed temperatures of 200 °C and above) and thus modulates the thermal emission from the semiconductor surface. As the excited carriers recombine (Fig. 1c), the modulation of the thermal emission redshifts in wavelength and decays in intensity with a time scale corresponding to the carrier recombination time in that semiconductor (~ns in GaAs and ~μs in Si). Thus, the total thermal emission profile following the short wavelength optical excitation consists of an ultrafast (~ps) short-wavelength pulse from the highly energetic hot carriers, followed by a longer time scale (~ns or ~μs) and increasingly longer wavelength emission from the modulation of the semiconductor’s baseline thermal emission via the free carriers’ effect on the semiconductor permittivity.

**Fig. 1:**
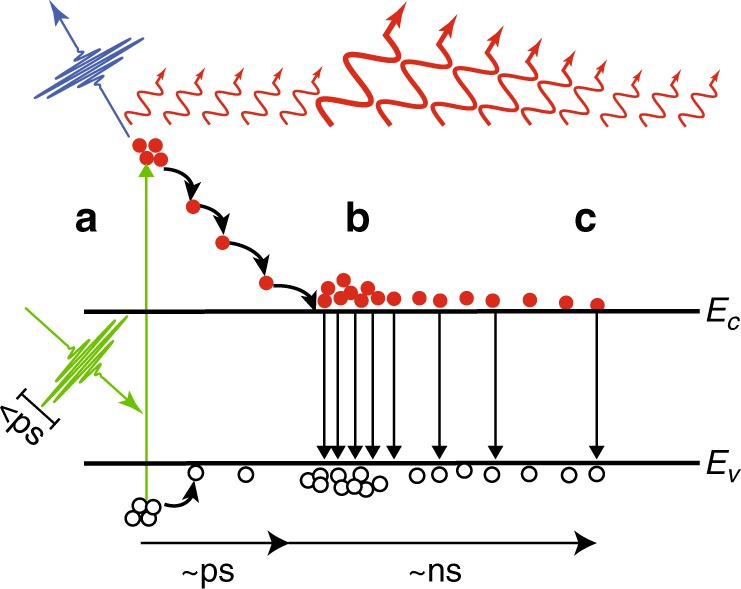
Schematic depiction of the temporal evolution of thermal emission in photoexcited intrinsic semiconductors. **a** A sub-ps pulse illuminates the semiconductor, generating highly energetic hot carriers that emit short-wavelength thermal emission (blue). **b** The hot carriers fall back to the band edge on ~ps timescales, at which time they modulate the thermal emission from the semiconductor lattice (red). **c** The modulation of the thermal emission then decays as the photoexcited carriers recombine on ~ns to ~μs times scales, depending on the semiconductor material

Over a series of careful experiments, the authors demonstrate the spectral and temporal evolution of the thermal emission resulting from this modulation of the permittivity and show indirect evidence for ultrafast thermal emission from the hot carriers, which is difficult to measure directly given the ~ps time scales associated with this effect. These results are supported by rigorous analytical models of the investigated material systems, which not only match the experimental data with great accuracy but also provide insight into the underlying physical mechanisms at play and the potential for greater spectral and temporal control of the thermal emission.

Thermal emission has long been recognized and utilized as a low-cost option for the generation of broadband mid-IR light. Most, if not all, mid-IR spectroscopy systems use glo-bars (essentially a highly emissive heated element) as their broadband IR source for reflection, transmission, and absorption spectroscopy studies. Recent work has examined mechanisms by which the broadband nature of thermal emission can be modified to create spectrally selective mid-IR sources. However, in the vast majority of demonstrated thermal emitters, it has been assumed that emission is largely constant or at best modulated only on millisecond time scales. The ability to tune a thermal emitter’s temperature on fast timescales remains exceedingly difficult due to the time constraints associated with the dissipation of thermal energy from any macroscale emitting surface. What Qiao and colleagues demonstrate is that a material’s emissivity can be controlled at much faster time scales than its lattice temperature. Thus, control of thermal emission is no longer limited by the long time scale of bulk thermodynamics but by the microsecond and nanosecond timescales of optoelectronic phenomena (in this case, carrier recombination).

With the ability to engineer carrier dynamics in semiconductor materials, as well as well-established mechanisms for spectral and spatial control of thermal emission, this work marks only the beginning of the investigation into ultrafast thermal emitters and their potential applications, offering the opportunity for the design and development of an entirely new class of infrared emitter.
